# Exploring dengue genome to construct a multi-epitope based subunit vaccine by utilizing immunoinformatics approach to battle against dengue infection

**DOI:** 10.1038/s41598-017-09199-w

**Published:** 2017-08-23

**Authors:** Mudassar Ali, Rajan Kumar Pandey, Nazia Khatoon, Aruna Narula, Amit Mishra, Vijay Kumar Prajapati

**Affiliations:** 1 Department of Biochemistry, School of Life Sciences, Central University of Rajasthan, Bandarsindri, Kishangarh, Ajmer, 305817 Rajasthan India; 2 Cellular and Molecular Neurobiology Unit, Indian Institute of Technology Jodhpur, Jodhpur, India

## Abstract

Dengue is considered as a major health issue which causes a number of deaths worldwide each year; tropical countries are majorly affected by dengue outbreaks. It is considered as life threatening issue because, since many decades not a single effective approach for treatment and prevention of dengue has been developed. Therefore, to find new preventive measure, we used immunoinformatics approaches to develop a multi-epitope based subunit vaccine for dengue which can generate various immune responses inside the host. Different B-cell, T_C_ cell, and T_H_ cell binding epitopes were predicted for structural and non-structural proteins of dengue virus. Final vaccine constructs consisting of T_C_ and T_H_ cell epitopes and an adjuvant (β-defensin) at N-terminal of the construct. Presence of B-cell and IFN-γ inducing epitopes confirms the humoral and cell mediated immune response developed by designed vaccine. Designed vaccine was not found allergic and was potentially antigenic in nature. Modeling of tertiary structure and the refined model was used for molecular docking with TLR-3 (immune receptor). Molecular docking and dynamics simulation confirms the microscopic interactions between ligand and receptor. *In silico* cloning approach was used to ensure the expression and translation efficiency of vaccine within an expression vector.

## Introduction

Dengue is one of the major public health concerns for people living in tropical and sub-tropical regions of the world; where approximately one-third population of the world, living under the risk of dengue infection^1^. There is a drastic increase of dengue infection around the world in past few decades. According to the World Health Organization (WHO) report, 390 million new cases occur each year worldwide^2^. A total of 100 countries in Asia, Pacific, America, Africa, and the Caribbean remain affected by dengue fever^3^. According to National Vector Borne Disease Control Programme (NVBDCP), dengue cases in India have been increased by 5 fold in last 5 years^4^. Dengue is caused by the transmission of any one of the four viral strains namely DEN-1, DEN-2, DEN-3, and DEN-4, belonging to the *Flaviviridae* family. The genome of *Flaviviridae* family members consisting of a positive-sense single-stranded RNA. *Flavivirus* and *Hepacivirus* are two genera come from the *Flaviviridae* family. Yellow fever virus (YFV), dengue fever virus (DENV), West Nile virus (WNV), Japanese encephalitis virus (JEV) are some important human pathogen belongs to *Flavivirus* genus while hepatitis C virus (HCV) belongs to *Hepacivirus*. The viral infection of dengue is being spread by the bite of female mosquitoes namely *Aedes aegypti and Aedes albopictus* ^2^. WHO classified dengue fever into two different groups, i.e.; uncomplicated and severe dengue fever. The symptoms associated with viral infection are high fever, headache, skin rashes, nausea, vomiting, muscle and joint pains, pain behind the eyes and these symptoms last for about 2–7 days. Severe symptoms like severe abdominal pain, red patches on skin, bleeding gums, thrombocytopenia (platelet count, ≤100,000/mm^3^), plasma leaking, fluid accumulation, difficulty in breathing, clammy skin, blood in vomit, drowsiness and black, tarry stool are associated with the severe dengue fever also called as dengue hemorrhagic fever (DHF)^1, 5^.

Dengue virus (DEN) genome consists 10,696 nucleotides of which 10,173 nucleotides encodes for the single open reading frame which corresponds to 3,391 amino acids^6^. DEN is single stranded positive sense RNA virus, the viral genome encodes for structural (Capsid protein C, precursor membrane protein, prM and envelope protein E) and non-structural proteins that include NS1, NS2B, NS2A, NS4A, NS3, NS4B, and NS5. Capsid protein or protein C has a role in virus budding and fusion with the membrane and helps in the formation of the core by assembling viral RNA in nucleo-capsid^7^. Peptide pr inhibits early fusion of envelope proteins by binding to envelope protein E in trans-Golgi at pH 6.0^8^. Protein prM masks and inactivates envelope protein E fusion peptide, it is the only viral protein matured in trans-Golgi network^9^. Small envelope protein M has a viroporin activity^10^, and its function is budding of virus^11^. Envelope protein E mediates host and viral membrane fusion^8^. NS1 involved in the development of infection by evading the host immune system and viral replication, it is being secreted as hexameric lipo particle which involves in immune evasion^12^. NS2A involved in viral replication and assemble viral particle, it also neutralizes the host immune response against virus^13^. NS2B act as a cofactor for NS3 for its serine protease activity, NS2B may have membrane destabilizing activity which alters the membrane permeability^14^. NS3 has an enzymatic activity, which are a serine protease, RNA helicase, and NTPase^15–17^. NS3 and NS2B complex involved in the autocatalytic cleavage of the polyprotein, RNA helicase activity of NS3 unwinds double-stranded RNA in direction of 5′ to 3′^16^. NS4A is not associated with the 2k fragment and it involves in the alteration of the cytoplasmic membrane, it also acts in the regulation of ATPase activity of NS3 helicase^18^. Peptide 2k acts as a signal sequence which involves in the translocation of NS4B into ER lumen^18^. NS4B involves in the inhibition of interferon signaling by blocking the promoters, STAT1 and interferon-stimulated response element (ISRE) activation^19^. NS5 has RNA-directed RNA polymerases activity, it involves in viral replication and capping. NS5 has a methyl-transferases activity which methylates the N7 of guanine and ribose at 2ʹO position^20^. NS5 also inhibit the activation of hosts JAK-STAT signaling pathway by blocking the activation of interferon α/β^21^. Apart from this, if we consider the evolutionary pattern of Dengue virus serotype diversification, it took around 200 years to make 4 viral lineages. This statement stating that the process of genetic diversity is slower that can be neglected if there is a chance of sudden benefit for the human being.

Till date, there is no preventative treatment or targeted therapies available for dengue fever, although proper intensive care and careful clinical administrations can help a lot to treat dengue. Vaccines not only provide immunity against pathogen but also considered as an effective way to control vector-borne infectious diseases. It can be used to prevent drug based therapy because there is a chance of toxicity associated with drug-based therapy. Immunoinformatics offers *in silico* approach which is being used for the development of a stable and multi-epitope vaccine in a short period of time and is considered as a cost-effective method than the traditional approach for the vaccine development in the laboratory. First dengue vaccine was developed in 1929^22, 23^ since then various vaccines were undergone clinical trials but none of them shows effectivity against the pathogen. A recent research shows the evolution of live attenuated tetravalent dengue vaccine namely Dengvaxia. Dengvaxia was the first dengue vaccine developed by Sanofi Pasteur. For the first time, it was approved in Mexico in December 2015 to cure the people between the age group of 9–45 years living in endemic zones for use in people 9–45 years old living in endemic zones. But, its recombinant live attenuated nature creates a high risk of reversal of attenuated vaccine strain into the more lethal viral strain. Apart from this, clinical trials of Dengvaxia show that it is not much effective against the DENV-2 strain. Current vaccine trials result shows that many individuals suffering from serious potential allergic reactions like asthma, urticaria, and headache. Additionally, clinical trial results of Dengvaxia shows adverse effects on a pregnant woman which results in abortion, stillbirth, elective termination, and death in the uterus. This situation urges the necessity of a new vaccine candidate that should be effective against the DENV-2 strain of dengue virus. Therefore, in this research work, we tried to design a novel multi-epitope subunit vaccine to terminate the chances of virulence reversal and strengthen robust innate, humoral and cell-mediated immunity by a combination of B cell, T cell, and IFNγ epitopes. The major advantages associated with the multi-epitope subunit vaccine is, the absence of any live component that reducing the associated risk of disease. Also, production of this vaccine does not require any pathogen involvement as these are simple peptide sequences which can be produced at the laboratory level. Protein production and purification technology can be applied to get the pure form of proposed vaccine constructs. By utilizing recent technological applications for protein purification we can separate the desired protein part from non-protein of the mixture. The first step of protein purification included preparation of crude extract followed by purification of the protein from crude extract by precipitation method. Further purification and assessment can be performed by utilizing chromatographic and blotting techniques.

Moreover, we analyzed the genome sequence of DEN-2 and retrieve the structural and non-structural protein sequence from UniProt. Different structural and non-structural proteins sequence were utilized for the prediction of CTL, HTL and B-cell epitopes. CTL and HTL epitopes were selected on the basis of their shared sequences on B-cell epitopes, to enhance the immunogenic response of vaccine, a small stretch of amino acid sequence from β-defensin was added at the N-terminal end of the final construct which will act as adjuvant^24^. After addition of an adjuvant, the final sequence was again used for prediction of B-cell and interferon-γ (IFN- γ) binding epitopes. The construct was further analyzed to evaluate allergenicity and antigenicity, also ExPASy–ProtParam tool was used to examine the various physiochemical properties (molecular weight, half-life, sequence length, aliphatic index, instability index, theoretical pI, and grand average of hydropathicity). The secondary structure of vaccine construct was also modeled using PsiPred, while homology modeling was performed for the prediction of tertiary structure. The predicted tertiary structure was further analyzed for its allowed and disallowed regions in Ramachandran plot by RAMPAGE. After generation of the tertiary structure of vaccine, molecular docking of vaccine structure with TLR-3 was assessed in order to examine interactions between ligand and receptor molecule. Docked complex was further analyzed in molecular dynamics simulation to validate stable interaction between ligand and receptor at the microscopic level. *In silico* cloning approach was performed to ensure the translation and expression efficiency of vaccine inside an expression vector.

## Results and Discussion

### Viral protein selection for vaccine preparation

For the construction of candidate vaccine, different structural and non-structural protein sequences of the DEN-2 virus were retrieved from UniProt. Protein C, prM and envelope proteins were the structural proteins of the virus, while NS1, NS2B, NS2A, NS4A, NS3, NS4B, and NS5 are non-structural proteins. Structural protein helps virus in host invasion and assembly of viral particles, whereas non-structural proteins secrete different enzymes which help in the viral replication and synthesis of structural proteins. When entering inside host they induce various immune responses. Amino acid sequences of these proteins were analyzed further for the prediction of CTL and HTL epitopes.

### Cytotoxic T lymphocyte (CTL) epitope prediction

For each structural and non-structural protein CTL epitopes were predicted from NetCTL 1.2, the server provides a score to each epitope. A high score indicates high specificity but low sensitivity of epitope. A total of 60 CD8+ T cell epitopes were predicted for all structural and non-structural proteins altogether, although in some cases very less or no epitopes were predicted (Supplementary Table 1). Longer the peptide sequence, more will be the number of epitopes, therefore, 18 epitopes were predicted for NS-5 while 10 epitopes were predicted for NS-3, whereas no epitopes were predicted for capsid protein and for NS-2B only 1 epitope was predicted due to small sequence.

### Helper T lymphocyte (HTL) epitopes prediction

Helper T lymphocyte (HTL) epitopes for all structural and non-structural proteins were predicted from IEDB server for mouse MHC-II alleles (IAb, IAd, IAs, IEb, IEd and IEs). Top 10 Epitopes were selected based on their IC_50_ value, which should be less than 50 nM with least percentile rank, which depicts high affinity of epitopes. Final selection of epitopes was done on the basis of their overlapped sequences, overlapped epitopes were merged together to obtain final epitopes (Supplementary Table 2).

### Prediction of B-cell epitope

Linear B-cell epitopes were predicted from BCPREDS for all structural and non-structural proteins. Predicted epitopes were further shortlisted on the basis of prediction score. All epitopes with a score more than 0.8 were selected for each structural and non-structural proteins. A total of 67 epitopes were predicted for all proteins while only 59 epitopes got more than 0.8 prediction score and were selected for further analysis (Supplementary Table 3). Maximum 18 linear epitopes were predicted for NS-5, although no epitope was predicted for NS-4A while only 1 epitope was predicted for NS-2A whose score is 0.745 thus it was not selected for further analysis due to low prediction score.

### Multi-epitope vaccine sequence construction

**Figure 1:**
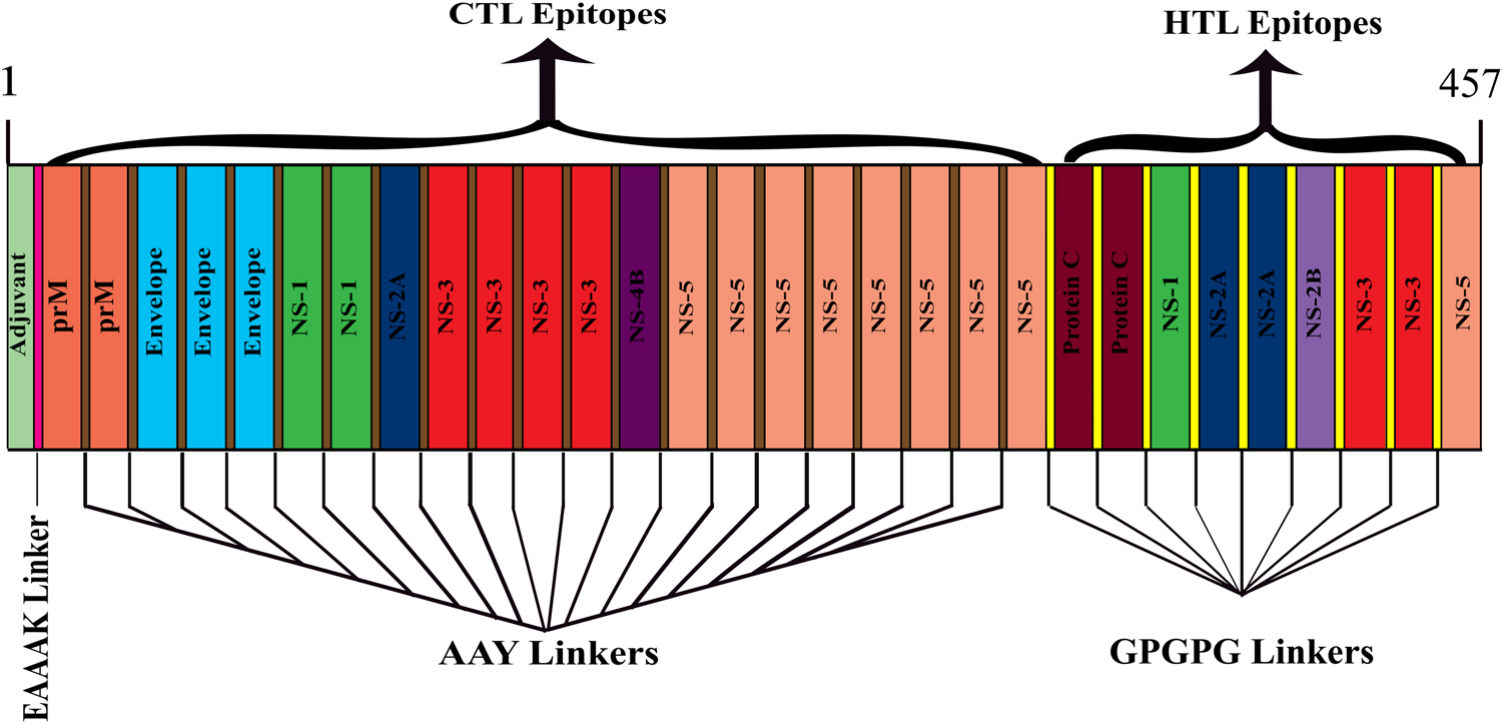
Schematic diagram of multi-epitope vaccine construct: A 457 amino acid long multi-epitope vaccine sequence consisting an adjuvant (green) at N-terminal end linked with a multi-epitope sequence with the help of EAAAK linker (pink). CTL epitopes were linked with the help of AAY linkers (brown) while HTL epitopes were fused with the help of GPGPG linkers (yellow).

For the construction of final vaccine sequence, predicted B-cell epitopes were treated as a template for CTL and HTL epitopes and those epitopes whose sequences were found to be overlapping in B-cell epitopes were shortlisted and selected for final vaccine construct (Table 1). A total of 16 CTL epitopes and 9 HTL epitopes were selected and merged by the help of AAY and GPGPG linkers respectively. After merging, the overall length of the construct was found to be 407 amino acids. Also, 45 amino acid long adjuvant (GIINTLQKYYCRVRGGRCAVLSCLPKEEQIGKCSTRGRKCCRRKK) was added at N-terminal of the construct by the help of EAAAK linker. After addition of linkers and adjuvant the final vaccine construct was found to be 457 amino acid long (Fig. 1).

**Table 1:** Selected CTL and HTL epitopes overlapping with B-cell epitopes.

Protein	B-cell Epitope	CTL epitope	HTL epitope
**Protein C**	TIPPTAGILKRWGTIKKSKA		QGRGPLKLYMALVAFLRFLTI
			WGTIKKSKAINVLRGFR
**prM**	NEPEDIDCWCNSTSTWVTYG	NSTSTWVTY	
	GLETRTETWMSSEGAWKHVQ		MSSEGAWKH
**Envelope**	HGTIVIRVQYEGDGSPCKIP	TTEAELTGY	
	GNDTGKHGKEIKITPQSSTT	VVQPENLEY	
	FRCKKNMEGKVVQPENLEYT	GTIVIRVQY	
	LTGYGTVTMECSPRTGLDFN		
**NS-1**	IDGPETAECPNTNRAWNSLE	NVHTWTEQY	WTEQYKFQPESPSKLASAIQKAHEEG
	TDNVHTWTEQYKFQPESPSK		WNSLEVEDY
**NS-2A**	VMVMVGATMTDDIGMGVTYL	MTDDIGMGV	RDLGRVMVMVGATMTD
			VTYLALLAAFKVRPT
**NS-2B**	KNDIPMTGPLVAGGPLTVCY		IPMTGPLVAGGPLTVCYV
**NS-3**	WDVPSPPPMGKAELEDGAYR	KVDAIDGEY	IFRKRRLTIMDLHPGAG
	DEEREIPERSWNSGHEWVTD	KAELEDGAY	GVLWDVPSPPPMGKA
	QTEKSIEDNPEIEDDIFRKR	VTDFKGKTV	
	RKTFDSEYAKTRTNDWDFVV	RTNDWDFVV	
	DNINTPEGIIPSMFEPEREK		
**NS-4B**	TVIDLDPIPYDPKFEKQLGQ	VIDLDPIPY	FLLVAHYAIIGPALQAKASREAQKRAAA
	AKASREAQKRAAAGIMKNPT		
**NS-5**	KGLTKGGPGHEEPIPMSTYG	YTDYMPSMK	
	EYTDYMPSMKRFRREEEEAG	LINRFTMRY	
	TKPWDVVPMVTQMAMTDTTP	RSLIGNEEY	
	AYHGSYETKQTGSASSMVNG	KTWAYHGSY	
	KIKQEHETSWHYDQDHPYKT	MTDTTPFGQ	
	FTMRYKKATYEPDVDLGSGT	ASSMVNGVF	
		MSTYGWNLV	
		QEHETSWHY

### Interferon γ (IFN- γ) inducing epitope prediction

**Figure 2:**
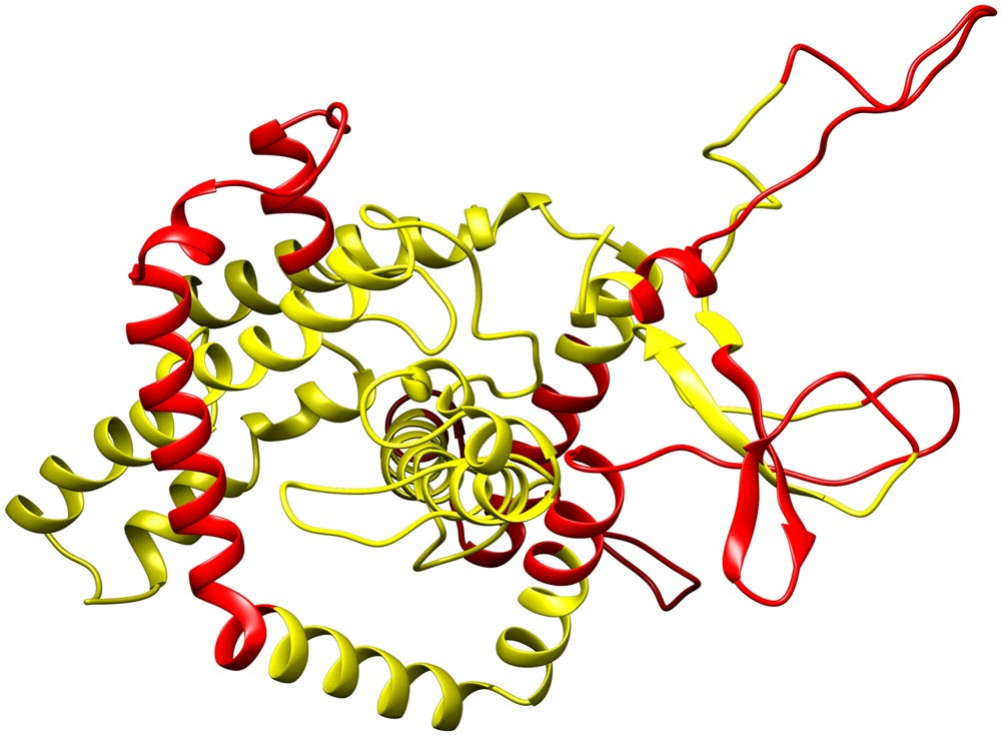
IFN-γ inducing and discontinuous B-cell epitopes representation in final vaccine model (**A**) Red color sequences depict IFN**-**γ inducing epitopes in the modeled structure. (**B**) Magenta color spheres show discontinuous B-cell epitopes in the 3D model of multi-epitope vaccine.

15-mer IFN-γ inducing epitopes were predicted for the final multi-epitope vaccine construct from IFNepitope server, it predicts epitope using MERCI software. A total of 399 epitopes were predicted having both negative and positive prediction scores out of which 58 IFN-γ inducing epitopes (Supplementary Table 4) carrying prediction score greater than 1 were selected (Fig. 2A).

### Allergenicity and antigenicity prediction

The AlgPred server was used to predict the non-allergic behavior of final vaccine construct. Prediction is based on SVM approach; it uses the amino acid sequence for prediction. Prediction result gives −0.40905 score which indicates that the vaccine construct is non-allergic in nature. A threshold score of prediction is −0.40. Prediction score less than threshold value point towards the non-allergic behavior of peptide. Antigenicity of vaccine construct was predicted using ANTIGENpro and followed by VaxiJen 2.0. The predicted antigenicity probability from ANTIGENpro was 0.830426 which shows a good antigenic nature of vaccine construct. Overall prediction for the antigen was performed by using VaxiJen, 0.6251 scores for overall prediction of antigen was obtained from VaxiJen, which indicates the construct is probable antigen. However, the threshold score of VaxiJen server is 0.4. Both results indicating that the final vaccine construct is a good antigen.

### Physicochemical analysis of vaccine constructs

Various physicochemical properties were calculated from ProtParam server. It uses an amino acid sequence of the vaccine construct for further analysis. The molecular weight of construct was calculated as 49 kDa which shows that construct has good antigenic properties. Initially, for the purpose of immunization, whole cells were used as vaccine candidate that was effective but some side effects were associated with the use of the whole cell as a vaccine. A well-known example is a vaccine against *Bordetella pertussis* that was developed and named as Acellular pertussis (aP) vaccine having multiple pertussis antigens namely pertussis toxin (Ptx), pertactin (Prn), fimbriae (Fim2 and Fim3), and filamentous hemagglutinin (FHA). Where the molecular weight of filamentous hemagglutinin (FHA) and pertactin (Prn) were 200 kDa and 69 kDa respectively^25^. Therefore the molecular weight of our vaccine may be good to initiate an immunogenic reaction. The analysis shows 8.86 pI (Isoelectric point) value which indicates the vaccine construct is slightly basic in nature. Estimated half-life in mammalian reticulocyte (*in vitro*) was found to be 30 hours, while in yeast and *Escherichia coli* the estimated half-life (*in vivo*) are >20 hours and >10 hours respectively. Estimated aliphatic index is 70.59 which indicates the protein is thermostable, high the value of aliphatic index more thermostable the protein. Grand average of hydropathicity (GRAVY) was calculated as −0.200, a negative value indicates the protein is hydrophilic in nature and it will interact with water molecules.

### Secondary structure prediction

**Figure 3:**
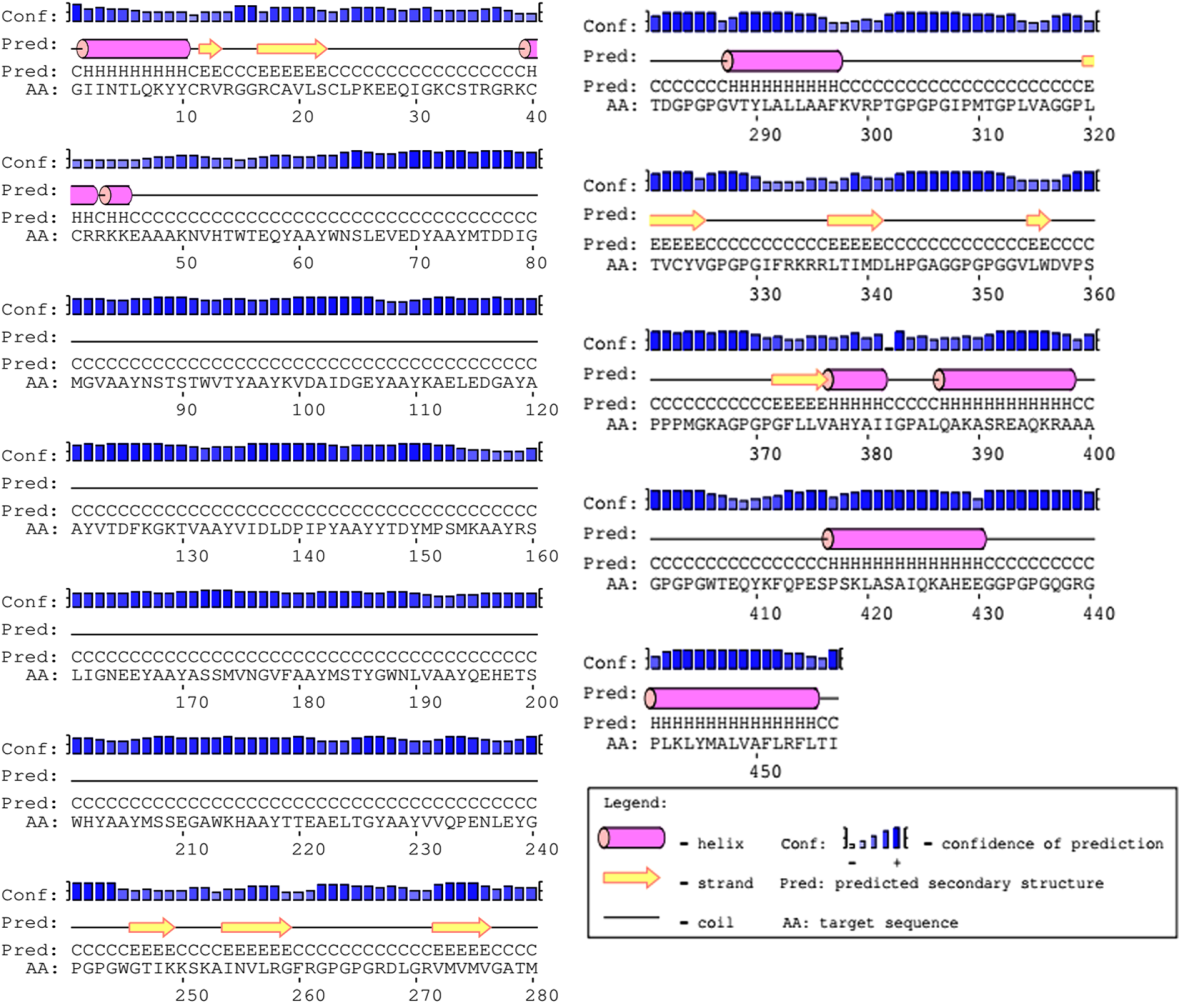
Schematic representation of secondary structure prediction of the multi-epitope vaccine, secondary structure prediction result represents the arrangement of α-helix (15.31%), β-strands (8.91%) and coils (75.71%).

The secondary structure of the final vaccine construct was predicted from online server PSIPRED. It predicts secondary structure on the basis of the amino acid sequence of the protein. 457 amino acid long construct was analyzed in which 346 amino acids involved in coil formation and 70 amino acids involved in α-helix formation while only β-strands are formed only by 41 amino acids. Overall secondary structure prediction result indicates 75.71% are coils, 15.31% are helix while 8.91% are stranded (Fig. 3).

### Tertiary structure modeling

**Figure 4:**
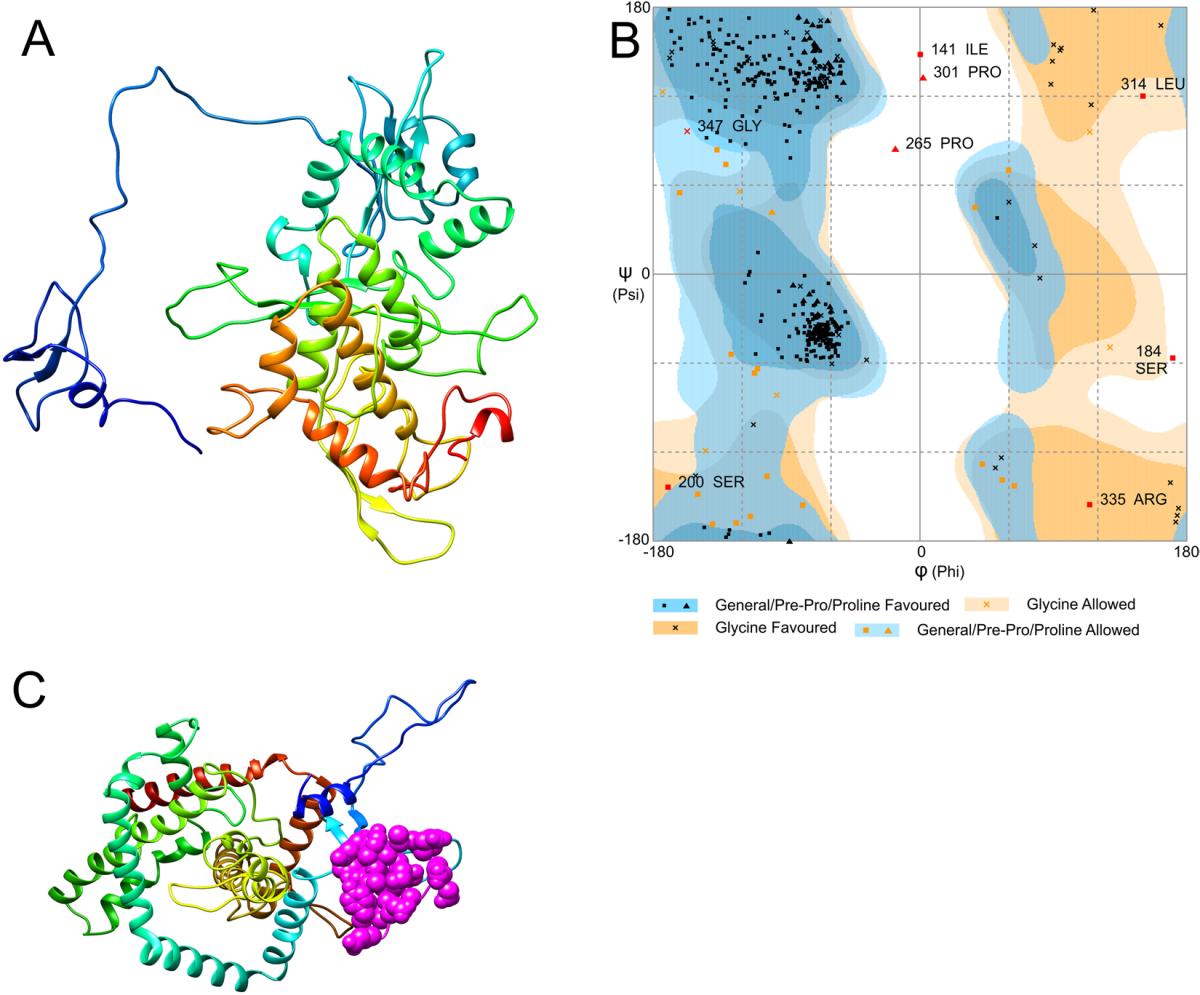
Multi-epitope vaccine modeling and validation (**A**) Represents tertiary structure of multi-epitope vaccine after modeling and refinement. (**B**) Shows validation of multi-epitope vaccine tertiary structure by ramachandran plot where 93% residues were found in favored region, 5.3% residues were found in allowed region and 1.8% residues were lies in outlier region.

The tertiary structure of multi-epitope vaccine was modeled from RaptorX server. It uses an amino acid sequence as an input data and input data was predicted to have two domains. 4c1oA was used as the best template for modeling. The relative quality of modeled structure was evaluated by P-value, calculated P-value for the modeled structure was 1.80 × 10^−3^ which is very low, lower the P-value higher the quality of the model. All 457 amino acids were modeled and 0% disorderedness was found in the model (Fig. 4A). Above findings can conclude as the tertiary structure model was highly ordered, stable and have high quality.

### Tertiary structure refinement

The predicted tertiary (3D) structure of final vaccine construct was further processed using GalaxyRefine server for model refinement. GalaxyRefine generates 5 models after refinement, out of which model 1 was selected due to its properties. The refined model shows 93% favored region in Ramachandran plot which is the best score among all the refined models. Also, GDT-HA was calculated as 0.9261, RMSD as 0.487, MolProbity as 2.396, clash score as 28.3 and poor rotamers as 0.6. Model 1 was found to be the best-refined model after comparison of different scores of it with remaining models (Supplementary Table 5), and it was selected for further studies.

### Validation of refined tertiary structure

Validation of refined tertiary structure was performed by using RAMPAGE server, which analyzes the structure and produces a Ramachandran plot for the input structure (Fig. 4B). Before refinement 88% structure came under favored region in plot, while 7.7% structure lies under allowed region and 3.7% structure came under disallowed region, while after refinement RAMPAGE generated better result, in which 93% structure was found to be in favored region, 5.3% came from allowed region and only 1.8% lies under disallowed region.

### Prediction of discontinuous B-cell epitope

Discontinuous B-cell epitopes were predicted using online web server namely ElliPro, which predict epitope on the basis of tertiary structure. Eight epitopes were predicted from the server from which epitope with maximum prediction score 0.903 was selected as a discontinuous epitope (Fig. 2B).

### Molecular docking of final vaccine construct with immunological receptor

**Figure 5:**
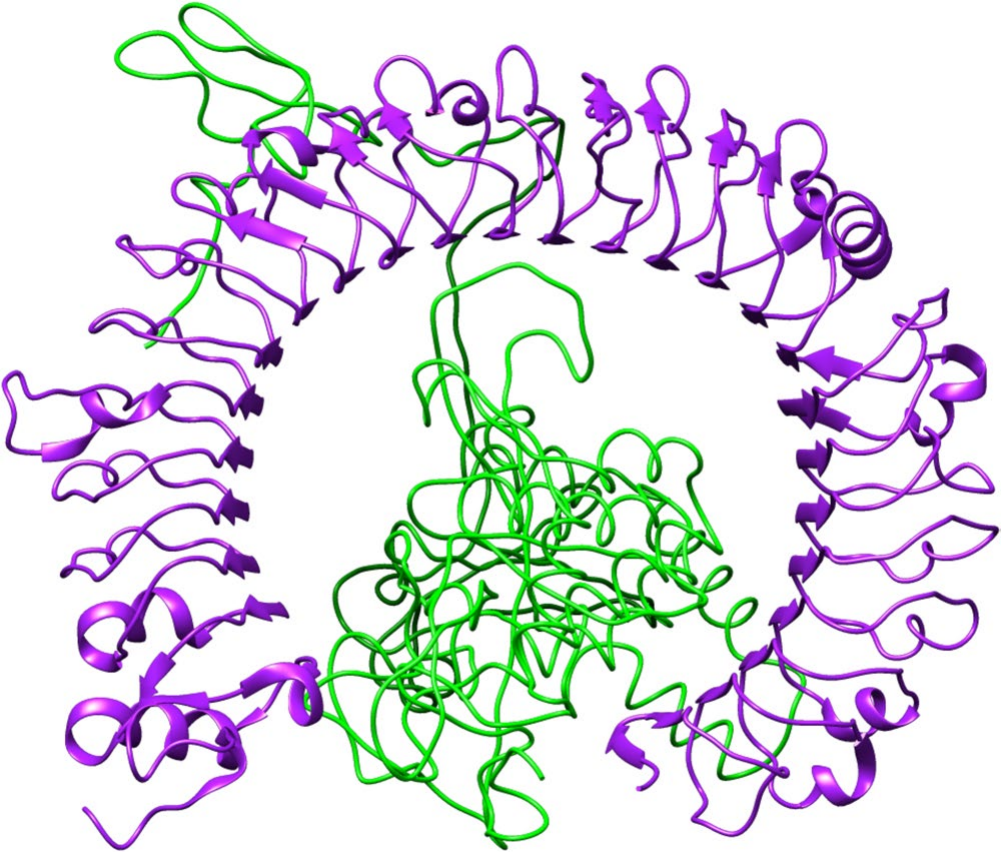
Ligand-receptor docked complex: figure obtained after molecular docking which represents multi-epitope vaccine as a ligand in green color while TLR-3 (PDB ID: 2A0Z) as a receptor in purple color.

Molecular docking was performed to evaluate the interaction between refined model and immune receptor TLR-3 (PDB ID- 2A0Z) by using online server PatchDock 4.0, was selected for protein-protein docking. PatchDock docking output gives multiple results out of which top 10 results were selected for analysis, after analyzing all 10 docked conformations, result number 2 was the best-docked model as it shows best interactions between receptor and ligand (Fig. 5). Docking score of geometric shape complexity was found to be 23122, calculated interface area of the interaction was 3392.20, three-dimensional transformation for second docked complex was estimated, in which translational parameters were calculated as −26.57, 29.54 and 39.13 and three angles for rotation was calculated as −1.98, 1.45 and −2.66. Also, atomic contact energy (ACE) of the complex was calculated as 75.52. Further analysis of docked complex was done by molecular dynamics.

### Molecular dynamics simulation of receptor-ligand complex

**Figure 6:**
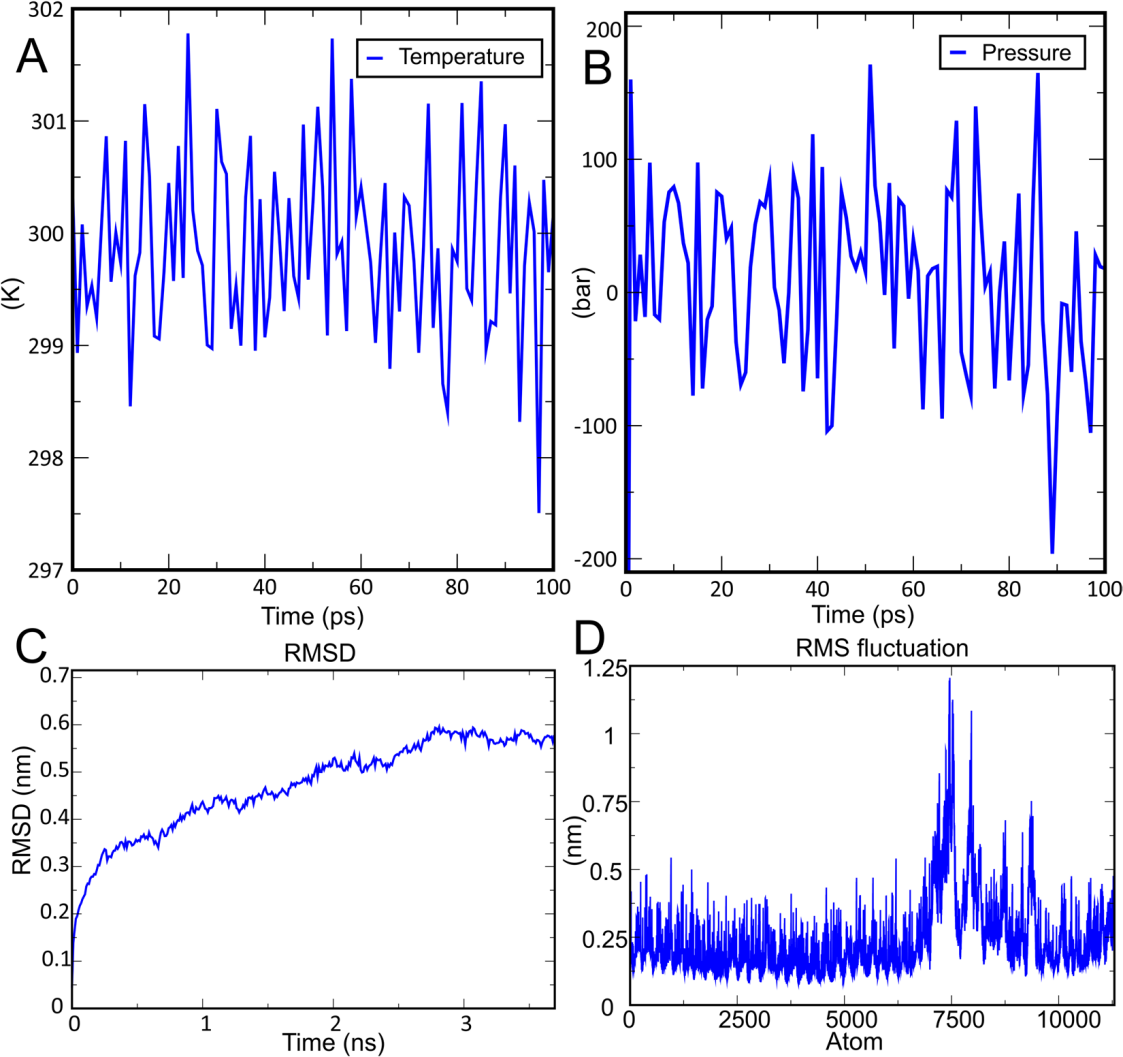
Molecular dynamics simulation of the ligand-receptor complex (vaccine & TLR-3) (**A**) Temperature progression plot of ligand-receptor complex shows that temperature of the system reaches to 300K and remains nearly constant around 300K throughout equilibration phase (100 ps). (**B**) Ligand-receptor complex pressure progression plot indicates fluctuation of pressure throughout the equilibration phase of 100 ps with an average pressure value 1 bar. (**C**) RMSD-Root Mean Square Deviation of docked complex shows very minute deviation which reflects the stable microscopic interaction between ligand and receptor molecule. (**D**) RMSF-Root Mean Square Fluctuation plot of docked protein complex side chain fluctuation in plot generates peak which reflects the flexibility of side chain of docked protein complex.

Molecular dynamics was performed for docked complex by using Gromacs 5.0 software to check stable interactions between ligand molecule (multi-epitope vaccine complex) and receptor (TLR-3) at the microscopic level. Energy minimization and calculation of pressure, temperature and potential energy were performed. The plot of temperature and pressure evaluation indicates that system maintains 300 K (Fig. 6A) for around 100 ps time interval and the system shows fluctuation in pressure with an average value 1 bar in the same time interval (Fig. 6B). To evaluate structural stability of ligand (multi-epitope vaccine complex) and receptor (TLR-3) complex root mean square deviation (RMSD) was observed. A plot of RMSD against time indicates, initially fluctuation generated with 0.15 nm and ends with 0.55 nm after 3.5 ns of the time interval, mild amount fluctuation reflects the stability of ligand-receptor complex over a period of 3.5 ns (Fig. 6C). The ligand-receptor interaction was further analyzed by the root mean square fluctuation (RMSF) of amino acid side chains (Fig. 6D). The plot shows very mild fluctuations in amino acid side chains which reflect the uninterrupted interaction between ligand and receptor whereas highly fluctuated regions in plot tell about highly flexible regions in the ligand-receptor complex. Higher peaks with an RMSF value of about 1.25 nm in the plot indicate highly flexible regions in the complex.

### *In silico* cloning

*In silico* cloning was performed by using online server Codon Usage Wrangler, in order to evaluate the cloning and expression of multi-epitope vaccine within the expression vector. Reverse translation generates cDNA sequence which was further analyzed by codon optimization analysis. Codon optimization evaluates the sequence and tells about GC content of the cDNA sequence and codon adaptive index (CAI) where GC content was calculated as 63.53% which lies in the optimum range (30–70%), and CAI was calculated as 1.00 which also lies in the range (0.8–1.0), high value of CAI indicates high expression of gene.

## Conclusion

Dengue has emerged as life threatening issue which has grown rapidly around the globe in past few decades, millions of cases of dengue were reported every year and cause the death of large population in tropical and sub-tropical countries. Statistical data indicates the seriousness of dengue infection; despite that, there is no preventative treatment or permanent therapy of dengue infection is currently available. Many attenuated vaccines for dengue were developed in recent years but none of them shows effectively. In this research, we utilized immunoinformatics approaches for the development of a multi-epitope vaccine against dengue virus (DEN virus). Development of multi-epitope vaccine does not require culturing of viral strain as it required in traditional vaccine development approaches. In order to provide humoral, innate and cell-mediated immunity multi-epitopes vaccine includes CTL and HTL epitopes which share common sequences with B-cell epitopes. CTL and HTL epitopes were merged together by using appropriate linker sequences. Generated vaccine model was further studied for prediction of IFN-γ inducing epitopes and discontinuous B-cell epitopes, also antigenicity and allergenicity of the sequence was predicted. In order to study stable interactions between ligand (multi-epitope vaccine) and receptor (TLR-3) molecular docking was performed and to molecular dynamics simulation was performed to validate interactions in the ligand-receptor complex. Finally, *in silico* cloning was performed to ensure expression and translation efficiency of the vaccine within appropriate expression and cloning vector.

## Methodology

### Dengue proteins sequence search for antigen prediction

In the first stage of vaccine development, genome of dengue virus (DEN) was studied through literature^26^ and to study different proteins of DEN virus, amino acid sequence of all proteins of strain DEN-2 including structural (UniProt ID- P29990) and non-structural (UniProt ID- P29990) viral proteins were retrieved in FASTA format from UniProt (Universal Protein Resource) database (http://www.uniprot.org/uniprot). Capsid protein, prM and envelope protein are structural proteins of DENV while NS1, NS2B, NS2A, NS4A, NS3, NS4B, and NS5 are non-structural proteins. β-defensin which is an agonist of Toll-like receptor 3 (TLR3) sequence was also retrieved in FASTA format. Prediction of antigenic epitopes is valuable for the examination of body self-defence system and help in vaccine component design. Antigenic epitopes are specific region present on the surface of the protein that is specially recognized by B-cell antibodies. All retrieved protein sequences were subjected to the antigen prediction that will be recognized by specific antibodies. Both, B-cell and T-cell antigens were predicted to enhance both humoral and cell-mediated immunity.

### Helper T Lymphocytes (HTL) epitope prediction

IEDB (http://tools.iedb.org/mhcii/) server was utilized to predict the T helper cell (HTL) epitopes of 15-mer length for all the structural and non-structural proteins. All epitopes were predicted for mouse MHC class II alleles (IAb, IAd, IAs, IEb, IEd and IEs). Prediction of MHC II epitopes was based on IC_50_ values and percentile rank; a peptide having highest affinity have IC_50_ values < 50 nM, peptides with intermediate affinity have IC_50_ values < 500 nM while peptides having IC_50_ value < 5000 nM shows the least affinity, therefore least IC_50_ value shows highest affinity^27^. Along with IC_50_ value of peptides, a percentile rank is developed by comparing the IC_50_ values of peptides against a set of random antigen from SWISSPROT database, compounds with least percentile rank show high affinity^28^.

### Cytotoxic T Lymphocytes (CTL) epitope prediction

For designing a vaccine, it was essential to predict cytotoxic T lymphocyte (CTL) epitopes. A freely accessible web server namely NetCTL 1.2 was utilized to predict the CTL epitopes for all structural and non-structural proteins. Prediction involves a combination of three approaches, MHC class-I binding affinity, TAP transport efficiency and proteasomal C-terminal cleavage. CTL epitope prediction was restricted by 12 MHC class-I super types^29^. MHC class-I peptide binding epitope prediction was attained by the use of artificial neural networks while TAP transport efficiency was achieved by an approach based on weight matrix^30^, while C-terminal proteasomal cleavage site was predicted by the artificial neural network from NetChop 3.1 server. Scores of all three predictions were merged together and sensitivity or specificity values can be achieved by the conversion of the threshold from the merged score. During CTL epitope prediction 0.75 was set as a threshold value for epitope identification.

### B-cell Epitope predictions

B-cell epitopes were predicted in order to evoke the high expression of antigen specific antibody production in the serum. In natural condition, antibody production starts with the encounter of immunogenic epitopes with the B cells receptor, leads to the differentiation into a plasma cell and a memory cell. In our immune system, plasma cell is responsible for the antibody production during primary infection while during secondary infection memory cells also help in antibody production. Prediction of linear B-cell epitopes for all the structural and non-structural proteins of DEN-2 was achieved by using online server BCPREDS: B-cell Epitope Prediction Server (http://ailab.ist.psu.edu/bcpred/predict.html). Epitope prediction by BCPREDS is based on three methods (i) AAP method^31^ (ii) BCPred^32^ and (iii) FBCpred^33^. AAP is amino acid pair antigenicity; this method is based on the fact that B-cell epitopes show particular AAPs using support vector machine (SVM) classifier. BCPred based on five different kernel methods having fivefold cross-validation by SVM. FBCpred utilizes subsequent kernel for the prediction of linear length B-cell epitopes. BCPred prediction method performed better (AUC 0.758) in comparison to AAP (AUC 0.7) method. The cutoff score of BCPreds is >0.8 for prediction of linear B-cell epitopes^34^.

### Construction of multi-epitope vaccine sequence

All different HTL and CTL epitopes, those were predicted by various immunoinformatic approaches were analyzed and scanned for an overlapping sequence with linear B-cell epitopes. All the overlapped epitopes of HTL and CTL were linked together with the help of GPGPG and AAY linkers respectively^35^. The β-defensin amino acid sequence was added as an adjuvant which linked at the N-terminal of above construct with the help of EAAAK linker^35^. Naïve T-cell and immature dendritic cells were recruited by β-defensin at the site of infection through the CCR6 receptor, and provide an adaptive immune response, β-defensin also provide innate host response in microbial infections^24^. Insertion of linkers between two epitopes provides efficient separation which is required for the effective functioning of each epitope^36^.

### IFN-γ inducing epitope prediction

Interferon gamma (IFN-γ) plays an important role in adaptive and innate immune response by stimulating macrophages and natural killer cells and provides a heightened response to MHC antigens. IFN-γ epitopes were predicted from IFNepitope server (http://crdd.osdd.net/raghava/ifnepitope/scan.php). The server basically constructs overlapped sequences, which is used for IFN-γ epitope prediction. The prediction was performed by motif and support vector machine (SVM) hybrid approach. The server is based on a dataset which consists IFN-γ inducing and non-inducing MHC class-II binder, which can activate T-helper cells^37^.

### Allergenicity and antigenicity prediction

The non-allergic or allergic behavior of multi-epitope vaccine construct was predicted by using a freely accessible online tool namely AlgPred (http://www.imtech.res.in/raghava/algpred/). Allergen prediction is based on similarity of known sequences of protein. IgE mapping of epitopes, which can locate the epitope position in the given protein. AlgPred uses MAST to search MEME/MAST allergen motifs and predict the allergen if it has a motif. It is an SVM module based program which uses amino acid or dipeptide composition for the prediction of allergen. It also performs BLAST against 2890 ARPs (allergen-representative peptides); it listed out the allergen protein if it has a BLAST hit. AlgPred uses all these parameters (IgE epitope + MAST + SVM + ARPs BLAST) combinedly to predict the allergenicity of peptide^38^.

To check whether multi-epitope vaccine sequence is antigenic or not, its antigenicity was predicted by online server ANTIGENpro (http://www.scratch.proteomics.ics.uci.edu/), this web server is based on the input sequence, free from any alignment and does not depend on any pathogen identity for the prediction of antigenicity. Prediction is a two-step process which is based on five algorithms and multiple representations of the sequence. A summarize result of prediction was generated by SVM classifier which tells about the probability of a peptide carrying characteristics of antigen^39^. To reconfirm the antigenicity of constructed multi-epitope vaccine, we also analyzed the antigenic property using VaxiJen server (http://www.ddg-pharmfac.net/vaxijen/VaxiJen/VaxiJen.html). Prediction of antigen in VaxiJen is alignment-free and based on various physiochemical properties of protein^40^.

### Prediction of various physicochemical properties

We further analyzed the multi-epitope vaccine for their various physicochemical properties which include molecular weight, half-life, sequence length, aliphatic index, instability index, theoretical pI, and grand average of hydropathicity by using of ProtParam tool (http://web.expasy.org/protparam/)^41^. ProtParam is a bioinformatics tool which can compute various physicochemical properties on the basis of pK values of different amino acids^42^. *In vivo* half-life prediction by ProtParam is based on the ‘N-end rule’ which states that the degradation of protein is determined by the N-terminal amino acids^43^. Instability index predicts whether a protein is stable or unstable, if the predicted instability index of a protein is below 40 then it’s called stable and proteins with the value above than 40 are comes under the category of unstable proteins. The volume occupied by the aliphatic side chains (alanine, valine, leucine, and isoleucine) is known as the aliphatic index of protein. Grand Average of Hydropathy was calculated by the sum of hydropathy obtained for all of the amino acid residues divided by the total number of amino acid residues present in the protein.

### Secondary structure prediction

PSIPRED server (http://bioinf.cs.ucl.ac.uk/index.php?id=779) was utilized to predict the secondary structure of the multi-epitope vaccine protein. PSIPRED is based on two feed-forward neural networks, where the initial prediction was achieved by the first network and secondary structure predicted by PSIPRED was used as input for the second network, which executes a study on output generated from PSI-BLAST (Position-Specific Iterated–BLAST) and refines the structure obtained from the first prediction^44^. An average Q_3_ score of PSIPRED 3.2 server is 81.6%.

### Tertiary structure prediction

The tertiary structure of final multi-epitope vaccine was predicted from web based server RaptorX (http://raptorx.uchicago.edu/StructurePrediction/predict/). RaptorX prediction is based on three components namely single-template threading, alignment quality prediction, and multiple-template threading. It is a template-based protein modeling tool which enhances the quality of alignment^45^. RaptorX generates high-quality tertiary structure models by multiple templates, the confidence score of the prediction gives an idea about the quality of predicted model. Confidence scores consist P-value and GDT (global distance test) for the relative global quality and absolute global quality respectively^46^.

### Tertiary structure refinement

The nature of protein model structure produced by contemporary protein structure prediction strategies firmly relies on the level of likeness between the input and available template structure. Therefore, there was a need of enhancing the template based protein model structure beyond the precision by refining the whole protein structure. Refinement of template based modeled tertiary structure was done by using freely accessible GalaxyRefine server (http://galaxy.seoklab.org/cgi-bin/submit.cgi?type=REFINE). This refinement method has the capability to improve both the global and local structural quality. Both mild and aggressive relaxation method was used to refine the whole protein model and generate the refined models with more structural deviation from the initial input structure. Refinement was achieved by rebuilding and repacking of side chains followed by subsequent overall relaxation and repeated structural perturbation by molecular dynamics simulation. The output of GalaxyRefine gives five structure models, in which model 1 was generated by the structural perturbation applied only to the clusters of side chains whereas model 2~5 was generated by more aggressive perturbations of loops and secondary structural elements. To avoid breakage in modeled structure by perturbation a tri-axial loop closure method was used^47^.

### Tertiary structure validation

Ramachandran plot was created by the use of online web server namely RAMPAGE (http://mordred.bioc.cam.ac.uk/~rapper/rampage.php) for the validation of tertiary structure of vaccine. Ramachandran plot is a visualization approach which predicts energetically allowed and disallowed dihedral angles psi (ψ) and phi (φ) of an amino acid; it is calculated on the basis of van der Waal radius of the side chain. RAMPAGE result includes the percentage of residues in allowed and disallowed regions which define the quality of modeled structure^48^.

### Discontinuous B-cell epitope prediction

B-cell discontinuous epitopes prediction of the vaccine was done by ElliPro (http://tools.iedb.org/ellipro/). ElliPro provides the score to each output epitope which described as PI (Protrusion Index) value averaged over each epitope residue. A number of ellipsoids approximated the tertiary structure of the protein. Ellipsoid with PI 0.9 value is considered as 90% protein residues are included while the remaining 10% residues lie outside of ellipsoids. For each epitope residues, the PI value is calculated on the basis of the center of mass of residue residing outside the largest possible ellipsoid. PI score is directly proportional to the solvent accessibility, greater the PI score more the solvent accessibility of the residues. The PI value of residues is the prediction criteria of discontinuous epitopes, while clustering is based on R (a distance between two residue’s center of mass, in Å). More prominent the value of R more will be the number of discontinuous epitopes predicted^49^.

### Molecular docking of vaccine with immune receptor

For a generation of the appropriate immune response, it is necessary for the antigenic molecule to interact with the immune receptor molecule. Molecular docking was performed to check the interaction between immune receptor (TLR-3) and ligand (multi-epitope vaccine). PatchDock (http://bioinfo3d.cs.tau.ac.il/PatchDock/) server was utilized to perform the molecular docking study of predicted vaccine protein and TLR-3 (PDB ID: 2A0Z) to show the immune responses, TLR-3 act as a receptor for antigenic response in case of dengue virus^50^. Object recognition and image segmentation techniques are used in the PatchDock algorithm where docking is inspired by assembling the jigsaw puzzle. During solving jigsaw puzzle it compares the sequences and finds complementary structures by superimposition of the structure using shape matching algorithm. Algorithm of PatchDock is divided into three different stages; the first one is molecular shape representation, followed by surface patch matching and filtering and scoring^51, 52^.

### Molecular dynamics simulation of receptor-ligand complex

To study the physical basis of function and structure of biological macromolecules molecular dynamics (MD) simulation was used^53–59^. To understand the structural properties and interaction between receptor (TLR-3) and ligand (predicted vaccine protein) at the microscopic level, molecular dynamics simulation study was performed by using Gromacs v5.0. GROMOS96 43A1 force field and the particle mesh Ewald summation method was used to run full system MD simulation by Gromacs. Pdb2gmx was used to construct protein topology, which gives an idea about bonded and non-bonded characteristics. Here, the PRODRG2 server was used to generate ligand topology. Neutralization of system was performed by the addition of sodium and chloride ions in required quantity. Energy minimization was performed prior to simulation to ensure that the geometry of the system is appropriate and there are no steric clashes by the use of steepest descent algorithm approach. System equilibration was performed in a two-step process, the first step is NVT while another step is NPT ensemble, both steps use leapfrog algorithm. In the system, during equilibration steps, the temperature was raised up to 300 K and pressure up to 1 bar. After completion of system equilibration, a 10 ns molecular dynamics simulation was attained for trajectory analysis^59^.

### *In silico* cloning

Reverse translation and codon optimization were performed from Codon Usage Wrangler (http://www.mrc-lmb.cam.ac.uk/ms/methods/codon.html) for cloning and its expression in the proper vector. Codon Usage Wrangler tool provides an output as cDNA sequence, which is further analyzed for codon optimization, codon adaptive index (CAI) and GC content by GeneScript Rare Codon Analysis Tool^60^. CAI tells about codon usage biases, ideal CAI score should be 1.0 but more than 0.8 can be considered as good score^61^. GC content of a sequence should range in between 30–70%, GC content values that do not reside in this range shows unfavorable effects on translational and transcriptional efficiencies. Lastly, EcoRI restriction site was added to the cDNA sequence.

## Electronic supplementary material

Supplementary Table
