# Supplementary material for: Exploring dengue genome to construct a multi-epitope based subunit vaccine by utilizing immunoinformatics approach to battle against dengue infection
Source: Sci Rep. 2017 Aug 23;7:9232. doi: 10.1038/s41598-017-09199-w (PMC5569093; doi:10.1038/s41598-017-09199-w)
Supplement: Supplementary file 1 — Supplementary Table [file 41598_2017_9199_MOESM1_ESM.pdf]

Exploring dengue genome to construct a multi-epitope based subunit vaccine by utilizing immunoinformatics approach to battle against dengue infection

Mudassar Ali[1], Rajan Kumar Pandey[1], Nazia Khatoon[1], Aruna Narula[1], Amit Mishra[2], Vijay Kumar Prajapati[1*]

[1]Department of Biochemistry, School of Life Sciences, Central University of Rajasthan, Bandarsindri, Kishangarh, Ajmer, 305817 Rajasthan, India.

[2]Cellular and Molecular Neurobiology Unit, Indian Institute of Technology Jodhpur, Jodhpur, India.

Supplementary table 1: Predicted CTL epitopes for structural and non-structural proteins of dengue virus

S. No.	Protein	Sequence	Score
1.	**prM**	TMMAAILAY	1.9915
2.		NSTSTWVTY	1.9773
3.		ELCEDTITY	1.1954
4.		MSSEGAWKH	0.9068
5.		TTEAELTGY	3.1769
6.		VVQPENLEY	2.1081
7.		QMSSGNLLF	1.3564
8.		QMENKAWLV	1.3366
9.		STSLSVTLV	1.2983
10.		VLVGIVTLY	1.0105
11.		GTIVIRVQY	0.9172
12.		KLQLKGMSY	0.8061
13.	**NS-1**	STESHNQTF	2.0681
14.		NVHTWTEQY	1.5861
15.		FTTNIWLKL	0.941
16.		ITDNVHTWT	0.929
17.		WNSLEVEDY	0.7769
18.	**NS-2A**	MTDDIGMGV	2.2165
19.		LTDALALGM	1.8278
20.		KTDWIPLAL	1.5206
21.		QVDNFSLGV	1.2211
22.		NMEKYQLAV	0.8604
23.		KMVRNMEKY	0.7628
24.	**NS-2B**	ITAAAWYLW	0.786
25.	**NS-3**	KVDAIDGEY	2.7669
26.		YSQIGAGVY	2.6923
27.		KAELEDGAY	1.5754
28.		FTDPASIAA	1.4073
29.		RVAAEGINY	1.2975
30.		YSDPLALKE	1.2756
31.		LSPVRVPNY	1.1299
32.		VTDFKGKTV	0.9561
33.		GVVTRSGAY	0.9323
34.		RTNDWDFVV	0.782
35.	**NS-4A**	ITASILLWY	2.9527
36.		TAEAGGRAY	1.3092
37.	**NS-4B**	VIDLDPIPY	1.7757
38.		LTAALFLLV	1.6802
39.		MANIFRGSY	1.4976
40.		YLAGAGLLF	1.1565
41.		ALFLLVAHY	0.7708

42.		TTLTAALFL	0.7589
43.	**NS-5**	YTDYMPSMK	2.2662
44.		ETACLGKSY	1.6863
45.		LINRFTMRY	1.6043
46.		RSLIGNEEY	1.5943
47.		FTDENKWKS	1.5825
48.		KTWAYHGSY	1.5116
49.		MTDTTPFGQ	1.2392
50.		YDQMWSLMY	1.1344
51.		KMEALQRKY	1.1153
52.		ASSMVNGVF	1.113
53.		MTTEDMLTV	1.1008
54.		LTSRATWAK	0.9451
55.		MSTYGWNLV	0.9043
56.		QEHETSWHY	0.8155
57.		FLEFEALGF	0.8038
58.		AEAIFKLTY	0.8019
59.		GSAKLRWFV	0.7776
60.		TTEDMLTVW	0.7598

Supplementary table 2: Predicted HTL epitopes for structural and non-structural proteins of dengue virus

S.No.	Protein	Start and end position	Epitope
1.	Protein C	39-59	QGRGPLKLYMALVAFLRFLTI
2.		69-85	WGTIKKSKAINVLRGFR
3.	prM	31-46	VNMCTLMAMDLGELCE
4.		85-100	EHRRQKRSVALVPHVG
5.		129-148	RHPGFTMMAAILAYTIGTTH
6.	Envelope	284-298	KCRLRMDKLQLKGMS
7.		406-422	MRGAKRMAILGDTAWDF
8.		439-465	VFGAIYGAAFSGVSWTMKILIGVIITW
9.	NS-1	28-53	WTEQYKFQPESPSKLASAIQKAHEEG
10.	NS-2A	51-66	RDLGRVMVMVGATMTD
11.		72-86	VTYLALLAAFKVRPT
12.		138-156	RNMEKYQLAVTIMAILCVP
13.	NS-2B	1-20	SWPLNEAIMAVGMVSILASS
14.		26-43	IPMTGPLVAGGPLTVCYV
15.	NS-3	2-16	GVLWDVPSPPPMGKA
16.		182-198	IFRKRRLTIMDLHPGAG
17.		261-279	CHATFTMRLLSPVRVPNYN
18.		437-453	GEERVILAGPMPVTHSS
19.	NS-4A	13-30	LPTFMTQKARDALDNLAV
20.		78-93	IGKMTLGMCCIITASI
21.		95-116	LWYAQIQPHWIAASIILEFFLI
22.	NS-4B	81-98	GWPLSKMDIGVPLLAIGC
23.		112-139	FLLVAHYAIIGPALQAKASREAQKRAAA
24.	NS-5	398-417	EFTRKVRSNAALGAIFTDEN
25.		609-626	LNTFTNMEAQLIRQMEGE

Supplementary table 3: Predicted B-cell epitopes for structural and non-structural proteins of dengue virus

S.No.	Protein	Position	Sequence	Score
1.	**Protein C**	58	TIPPTAGILKRWGTIKKSKA	0.998
2.		10	NTPFNMLKRERNRVSTVQQL	0.984
3.	**prM**	59	NEPEDIDCWCNSTSTWVTYG	0.998
4.		102	GLETRTETWMSSEGAWKHVQ	0.87
5.		4	TTRNGEPHMIVSRQEKGKSL	0.836
6.	**Envelope**	382	VEPGQLKLNWFKKGSSIGQM	1
7.		217	PLPWLPGADTQGSNWIQKET	0.994
8.		64	KLTNTTTESRCPTQGEPSLN	0.992
9.		353	TVNPIVTEKDSPVNIEAEPP	0.987
10.		317	HGTIVIRVQYEGDGSPCKIP	0.978
11.		152	GNDTGKHGKEIKITPQSSTT	0.976
12.		96	MVDRGWGNGCGLFGKGGIVT	0.914
13.		119	FRCKKNMEGKVVQPENLEYT	0.886
14.		175	LTGYGTVTMECSPRTGLDFN	0.869
15.		43	FELIKTEAKQPATLRKYCIE	0.849
16.		241	KNPHAKKQDVVVLGSQEGAM	0.813
17.		22	DIVLEHGSCVTTMAKNKPTL	0.803
18.	**NS-1**	105	RPQPTELKYSWKTWGKAKML	1
19.		293	NRGPSLRTTTASGKLITEWC	0.992
20.		325	GEDGCWYGMEIRPLKEKEEN	0.992
21.		135	IDGPETAECPNTNRAWNSLE	0.988
22.		243	IPKNLAGPVSQHNYRPGYHT	0.976
23.		22	TDNVHTWTEQYKFQPESPSK	0.877
24.		84	VKLTIMTGDIKGIMQAGKRS	0.851
25.	**NS-2B**	79	SEDGSMSIKNEEEEQTLTIL	1
26.		23	KNDIPMTGPLVAGGPLTVCY	0.947
27.	**NS-3**	569	NNQILEENVEVEIWTKEGER	1
28.		129	DFSPGTSGSPIIDKKGKVVG	0.999
29.		312	IFMTATPPGSRDPFPQSNAP	0.998
30.		102	PGKNPRAVQTKPGLFKTNAG	0.992
31.		78	SYGGGWKLEGEWKEGEEVQV	0.987
32.		5	WDVPSPPPMGKAELEDGAYR	0.978
33.		334	DEEREIPERSWNSGHEWVTD	0.965
34.		167	QTEKSIEDNPEIEDDIFRKR	0.928
35.		387	RKTFDSEYAKTRTNDWDFVV	0.923
36.		456	QRRGRIGRNPKNENDQYIYM	0.89
37.		496	DNINTPEGIIPSMFEPEREK	0.859
38.		527	GEARTTFVDLMRRGDLPVWL	0.855

39.		435	TDGEERVILAGPMPVTHSSA	0.826
40.	**NS-4B**	197	ATGPISTLWEGNPGRFWNTT	0.995
41.		151	TVIDLDPIPYDPKFEKQLGQ	0.846
42.	**NS-5**	101	KGLTKGGPGHEEPIPMSTYG	1
43.		878	EYTDYMPSMKRFRREEEEAG	0.998
44.		524	KKEGGAMYADDTAGWDTRIT	0.997
45.		575	NKVVRVQRPTPRGTVMDIIS	0.997
46.		819	QENPWMEDKTPVESWEEIPY	0.997
47.		144	LCDIGESSPNPTVEAGRTLR	0.993
48.		380	WLWKELGKKKTPRMCTREEF	0.974
49.		351	QQRVFKEKVDTRTQEPKEGT	0.964
50.		329	TKPWDVVPMVTQMAMTDTTP	0.96
51.		682	NDTGKIRKDIQQWEPSRGWN	0.958
52.		165	LNLVENWLNNNTQFCIKVLN	0.953
53.		20	LGKSEFQIYKKSGIQEVDRT	0.943
54.		304	AYHGSYETKQTGSASSMVNG	0.937
55.		457	KREKKLGEFGKAKGSRAIWY	0.936
56.		283	KIKQEHETSWHYDQDHPYKT	0.926
57.		781	SAVPSHWVPTSRTTWSIHAK	0.921
58.		243	FTMRYKKATYEPDVDLGSGT	0.891
59.		215	THEMYWVSNASGNIVSSVNM	0.807

Supplementary table 4: Interferon γ inducing epitopes for final vaccine construct predicted using IFNepitope server

S.No.	Start-End	Sequence	Score
1.	7—22	QYAAYWNSLEVEDYA	2
2.	8—23	YAAYWNSLEVEDYAA	2
3.	9—24	AAYWNSLEVEDYAAY	2
4.	10—25	AYWNSLEVEDYAAYM	2
5.	11—26	YWNSLEVEDYAAYMT	2
6.	12—27	WNSLEVEDYAAYMTD	2
7.	13-28	NSLEVEDYAAYMTDD	2
8.	14-29	SLEVEDYAAYMTDDI	2
9.	15-30	LEVEDYAAYMTDDIG	2
10.	16-31	EVEDYAAYMTDDIGM	2
11.	17-32	VEDYAAYMTDDIGMG	2
12.	18-33	EDYAAYMTDDIGMGV	2
13.	48-63	KVDAIDGEYAAYKAE	1
14.	49-64	VDAIDGEYAAYKAEL	1
15.	50-65	DAIDGEYAAYKAELE	1
16.	85-100	IDLDPIPYAAYYTDY	1
17.	86-101	DLDPIPYAAYYTDYM	1
18.	87-102	LDPIPYAAYYTDYMP	1
19.	88-103	DPIPYAAYYTDYMPS	1
20.	89-104	PIPYAAYYTDYMPSM	1
21.	90-105	IPYAAYYTDYMPSMK	1
22.	91-106	PYAAYYTDYMPSMKA	1
23.	92-107	YAAYYTDYMPSMKAA	1
24.	133-148	STYGWNLVAAYQEHE	1
25.	134-149	TYGWNLVAAYQEHET	1
26.	135-150	YGWNLVAAYQEHETS	1
27.	136-151	GWNLVAAYQEHETSW	1
28.	137-152	WNLVAAYQEHETSWH	1
29.	138-153	NLVAAYQEHETSWHY	1
30.	139-154	LVAAYQEHETSWHYA	1
31.	140-155	VAAYQEHETSWHYAA	1
32.	141-156	AAYQEHETSWHYAAY	1
33.	157-172	SSEGAWKHAAYTTEA	1
34.	158-173	SEGAWKHAAYTTEAE	1
35.	159-174	EGAWKHAAYTTEAEL	1
36.	160-175	GAWKHAAYTTEAELT	3
37.	161-176	AWKHAAYTTEAELTG	5
38.	162-177	WKHAAYTTEAELTGY	5

39.	163-178	KHAAYTTEAELTGYA	4
40.	164-179	HAAYTTEAELTGYAA	4
41.	165-180	AAYTTEAELTGYAAY	4
42.	166-181	AYTTEAELTGYAAYV	4
43.	167-182	YTTEAELTGYAAYVV	4
44.	236-251	GVTYLALLAAFKVRP	1.00042
45.	289-304	MDLHPGAGGPGPGGV	1.187181
46.	290-305	DLHPGAGGPGPGGVL	1.200201
47.	291-306	LHPGAGGPGPGGVLW	1.15008
48.	292-307	HPGAGGPGPGGVLWD	1.119006
49.	293-308	PGAGGPGPGGVLWDV	1.038983
50.	336-351	QAKASREAQKRAAAG	1
51.	337-352	AKASREAQKRAAAGP	1
52.	338-353	KASREAQKRAAAGPG	1
53.	352-367	GPGWTEQYKFQPESP	1
54.	353-368	PGWTEQYKFQPESPS	1
55.	355-370	WTEQYKFQPESPSKL	1
56.	356-371	TEQYKFQPESPSKLA	1
57.	357-372	EQYKFQPESPSKLAS	1
58.	358-373	QYKFQPESPSKLASA	1

Supplementary table 5: GalaxyRefine score of all predicted refined models

Model	GDT-HA	RMSD	MolProbity	Clash score	Poor Rotamer
**Initial**	**1.0000**	**0.000**	**3.646**	**128.7**	**4.1**
MODEL 1	0.9261	0.478	2.396	28.3	0.6
MODEL 2	0.9240	0.480	2.619	29.5	1.8
MODEL 3	0.9207	0.483	2.588	29.9	1.5
MODEL 4	0.9163	0.496	2.447	26.9	1.2
MODEL 5	0.9158	0.501	2.471	30.8	0.6
